# A Case Report of Anti-GAD65 Antibody-Positive Autoimmune Encephalitis Associated with Diabetes

**DOI:** 10.2174/0118715303405229251028051625

**Published:** 2025-12-04

**Authors:** Xiuping Xuan, Liheng Meng, Lulu Chen, Xuemei Xie, Xinghuan Liang, Yingfen Qin

**Affiliations:** 1 Department of Endocrinology, The First Affiliated Hospital of Guangxi Medical University, Nanning, Guangxi 530021, China;; 2 Department of Geriatric Endocrinology, The First Affiliated Hospital of Guangxi Medical University, Nanning, Guangxi 230021, China

**Keywords:** Glutamate decarboxylase 65, autoimmune encephalitis, diabetes, β cell function, hyperthyroidism, C-peptide

## Abstract

**Background:**

Patients with GAD65 antibody-associated autoimmune encephalitis who also have diabetes are usually considered to have type 1 diabetes mellitus (T1DM). However, previous reports often lacked β-cell function assessments, relying solely on anti-GAD65 positivity for diagnosis, which is insufficient for accurate classification.

**Case Presentation:**

We present a 30-year-old Chinese woman diagnosed with GAD65-associated autoimmune encephalitis, hyperthyroidism, and diabetes. Her peak C-peptide level was 3.99 ng/mL, inconsistent with the β-cell dysfunction typical of classic T1DM. The combination of high GAD65 antibody titers and preserved β-cell function made it challenging to determine her diabetes type.

**Conclusion:**

This case suggests that clinicians should pay more attention to assessing and monitoring β-cell function when GAD65-associated autoimmune encephalitis is accompanied by diabetes.

## 1 INTRODUCTION

Glutamate decarboxylase (GAD), an enzyme present in GABAergic neurons, is essential for synthesizing the inhibitory neurotransmitter GABA [1-3]. It exists as two isoforms, GAD65 and GAD67 [4], with GAD65 serving as the major autoantigen and being predominantly localized to synaptic terminals [5]. During exocytosis, GAD65 may be transiently exposed on the neuronal surface [6]. Anti-GAD65 antibodies have been associated with various neurological syndromes, including temporal/limbic epilepsy, ataxia, and Stiff-Person Syndrome [1, 2, 7]. These conditions are typically characterized by high titers of GAD65 antibodies in both serum and cerebrospinal fluid [5, 8]. Although controversial [9], GAD65 antibodies may induce neuronal damage *via* endocytosis, leading to intracellular antigen–antibody interactions [6]; however, other studies suggest that cytotoxic CD8^+^ T cells are the primary mediators of neuronal injury in GAD65-related limbic encephalitis [5, 10], with GAD65 antibodies acting mainly as biomarkers of T-cell–mediated damage [11]. The widespread distribution of GABAergic neurons in the hippocampus [12], cerebellum [12], basal ganglia [13], and spinal gray matter [14] may explain the characteristic clinical phenotypes: hippocampal dysfunction predisposes to temporal/limbic epilepsy [15]; cerebellar Purkinje cell impairment results in ataxia [16]; and reduced inhibitory control in the basal ganglia and spinal gray matter contributes to the muscle rigidity observed in Stiff-Person Syndrome [17, 18].

Patients with GAD65-associated neurological syndromes are often diagnosed with other systemic autoimmune disorders, such as type 1 diabetes mellitus (T1DM), thyroid disease, pernicious anemia, coeliac disease, and vitiligo [19, 20]. Among them, T1DM occurs in about half of the cases of GAD65-associated CNS syndromes [19]. Notably, as no records of β-cell function assessments were available in previous reports [1, 7, 20-22], the diagnosis of T1DM in patients with GAD65-associated autoimmune encephalitis might have been based solely on anti-GAD65 positivity. However, GAD65 antibody positivity alone is insufficient for the definitive classification of diabetes [23]. Clarifying the type of diabetes requires concurrent assessment of β-cell function, both at the time of diagnosis and during follow-up [24].

Here we present a 30-year-old woman diagnosed with anti-GAD65-mediated autoimmune encephalitis, hyperthyroidism, and diabetes. In this case, fasting and stimulated C-peptide levels following a standard 100-g steamed bun meal test were assayed to evaluate β-cell function. The peak C-peptide level was 3.99 ng/mL, which was inconsistent with the typically low stimulated C-peptide levels observed in classic T1DM [25]. This suggests to clinicians that when diabetes coexists with GAD65-associated autoimmune encephalitis, the diabetes might not necessarily be classic T1DM, and it is crucial to monitor β-cell function.

## 2 CASE PRESENTATION

We present a case with sequential diagnoses of hyperthyroidism, GAD65-associated autoimmune encephalitis, mesial temporal lobe epilepsy with hippocampal sclerosis (MTLE-HS), and diabetes mellitus. The following sections provide a detailed description of the patient’s clinical history. A timeline illustrating the chronological sequence of clinical and paraclinical findings is shown in Fig. (**1**). This case report was conducted in accordance with the ethical standards of the institutional review board and the principles of the Declaration of Helsinki. It was approved by the Medical Ethics Committee of the First Affiliated Hospital of Guangxi Medical University (Approval No. 2025-E0684). Written informed consent was obtained from the patient for publication of this report.

### 2.1 Hyperthyroidism History

The patient was diagnosed with hyperthyroidism at a local hospital in 2014, at the age of 20, following symptoms of palpitations and heat intolerance. The patient reported being treated with methimazole for 2 years, with the specific dosage unknown. Treatment was discontinued in 2017, and subsequent follow-ups showed normal thyroid function. No thyroid function test reports were available from 2017 to June 2023. Moreover, thyroid function tests for the patient conducted on November 28, 2023, were within the reference range (Table **1**).

### 2.2 Seizures History

In 2016, the patient was diagnosed with epilepsy at a local hospital after being unresponsive to attempts to be awakened from a nap. On March 26, 2017, a cranial MRI scan revealed a demyelinating lesion in the white matter of the left centrum semiovale (Figs. **2A** and **2B**). A brain Metabologram performed in 2021 showed reduced glucose metabolism in the right temporal lobe, which could not exclude an epileptic focus. No significant structural or glucose metabolism abnormalities were observed in other areas (Fig. **3**). From 2017 to 2023, the patient regularly visited the neurology outpatient clinic and was treated with lamotrigine, oxcarbazepine, and valproate sodium. However, during this period, the patient experienced generalized tonic-clonic seizures approximately once a year, absence seizures approximately four to five times per month, and significant memory impairment. In July 2023, the patient was hospitalized in the neurology department. Serum and cerebrospinal fluid (CSF) tests for GAD65 antibodies were performed, and both were positive at a titer of 1:100 (Table **2**). The patient was then diagnosed with: (1) GAD65-associated autoimmune encephalitis and (2) mesial temporal lobe epilepsy with hippocampal sclerosis (MTLE-HS). Subsequently, methylprednisolone pulse therapy was administered. After discharge, the treatment included sodium valproate sustained-release tablets, lamotrigine tablets, prednisone acetate tablets, clonazepam tablets, and levetiracetam tablets. Since then, there have been no further tonic-clonic seizures, but the frequency of absence seizures has remained the same as before. On October 26, 2023, a cranial MRI of the patient revealed white matter lesions, classified as modified Fazekas grade 1 (Figs. **2C** and **2D**). During follow-up, the CSF GAD65 antibody IgG test was positive with a titer of 1:100 on September 16, 2023, and the serum concentration was 1:300 on June 8, 2024 (Table **2**).

### 2.3 Diabetes History

In July 2023, during hospitalization in the neurology department, the patient had a 2-hour postprandial plasma glucose (2hPG) level of 24.27 mmol/L and a glycated hemoglobin (HbA1c) level of 16.40%, accompanied by symptoms of xerostomia. At that time, the patient was diagnosed with diabetes and was started on insulin aspart and insulin glargine to manage blood glucose levels. On August 11, 2023, the patient had a fasting blood glucose (FPG) of 17.8 mmol/L, a 2hPG of 7.68 mmol/L, a fasting C-peptide of 1.06 ng/mL, and a 2-hour postprandial C-peptide of 1.49 ng/mL (Table **3**). The blood glucose levels fluctuated significantly due to poor dietary control. On June 17, 2024, the patient consulted the endocrinology department. Diabetes education was reinforced, and intensive therapy was administered using an insulin pump. Simultaneously, serum GAD65 antibodies were assayed. The level of GAD65 antibodies was 631.8 IU/mL (Table **2**). Moreover, C-peptide levels were 0.13, 0.88, 2.62, 3.99, and 2.71 ng/mL after ingestion of a standard 100-g steamed bun meal (Table **3**). On the day of discharge, the FPG fluctuated between 5.7 and 7.3 mmol/L, and the 2hPG was around 6 mmol/L. The treatment regimen was reverted to multiple daily subcutaneous insulin injections to control blood glucose levels.

## 3 DISCUSSION

In this case report, the patient was diagnosed with GAD65-associated autoimmune encephalitis, with markedly elevated cerebrospinal fluid (CSF) GAD65 antibody (Ab) titers. The pathogenic role of these antibodies remains uncertain [9]. GAD65 is an intracellular antigen, raising doubt about its accessibility to circulating antibodies, and evidence for neuronal uptake of GAD65 antibodies is conflicting [9, 26]. Some studies have reported that incubation of primary hippocampal neurons with anti-GAD65 leads to its uptake by GABAergic neurons [26], enabling potential antibody–antigen interaction within the cytoplasm [6], whereas other researchers have found no such uptake [27]. Likewise, their effects on inhibitory synaptic transmission in central GABAergic pathways appear inconsistent: while incubation of primary hippocampal neurons with anti-GAD65 resulted in uptake by GABAergic neurons, neuronal cell death, and increased network activity [26], stereotactic injection of GAD65 antibody–positive human CSF into the rat hippocampus produced no detectable changes in either evoked or spontaneous GABAergic transmission [28, 29]. While these findings are contradictory, they suggest that GAD65 antibodies may have pathogenic potential, warranting further mechanistic investigation.

In addition to the potential role of antibodies, recent studies have highlighted the involvement of T-cell–mediated neuronal injury in GAD65-related epilepsy [5, 10]. T lymphocytes can impair neuronal function through a perforin-dependent “electrophysiological silencing” mechanism, producing neuronal dysfunction during central nervous system involvement [30]. Supporting this, increased activation of CD8^+^ T cells has been observed in both peripheral blood and CSF of patients with GAD65-related limbic encephalitis (GAD65-LE), with expression of perforin detected in peripheral blood and within the hippocampal parenchyma of patients with long-standing disease [5]. These findings implicate CD8^+^ T cells as potential promoters of autoimmune epilepsy–associated neurodegeneration in GAD65-LE. Furthermore, T cells recognize specific GAD65 antigen epitopes [31]. Interestingly, GAD65 antibodies in different neurological diseases recognize distinctly different epitopes. This disease-specific pattern of GAD65 antibody epitope recognition may explain why neurological diseases associated with GAD65 antibodies exhibit significantly different symptoms [32].

Generally, patients with GAD65 antibody-mediated autoimmune encephalitis who also have diabetes are typically considered to have T1DM [21, 22]. Similar to the ongoing debate about the pathogenic role of GAD65 antibodies in autoimmune encephalitis, their contribution to T1DM remains controversial. *In vitro* studies have shown that these antibodies can be internalized by islet cells and alter glucose-stimulated insulin secretion, suggesting a potential pathogenic effect [33]. However, other evidence indicates that GAD65 autoantibodies are not directly pathogenic, and may serve only as a marker of T1DM [34]; instead, T cells appear to play the central role in disease initiation and progression [35]. GAD65 associates with the plasma membrane and enters the extracellular space [36], where it is taken up by dendritic cells (antigen-presenting cells, APCs) *via* macropinocytosis or receptor-mediated endocytosis [36]. These APCs present GAD65-derived epitopes to CD4^+^ T cells *via* MHC class II and, through cross-presentation, to CD8^+^ T cells *via* MHC class I [5, 31]. In other words, only specific GAD65 epitopes are capable of being recognized by the corresponding T cells [5, 31]. Among recognized epitopes, some studies suggest that the HLA class I–restricted GAD65_114_–_123_ peptide is immunodominant in T1DM patients [5], whereas others suggest that the HLA class II–presented GAD65_271_–_285_ and GAD65_431_–_450_ peptides could be among the critical antigenic epitopes triggering the primary autoreactive responses in the early stages of the disease [35]. CD4^+^ T cells recognize class II epitopes, with activation occurring most commonly when presented by T1DM-associated alleles such as HLA-DR3, DR4, or DQ8 [35]. Once activated, CD4^+^ T cells provide help to B cells, promoting the production of autoantibodies (*e.g.*, anti-insulin), thereby accelerating T1DM progression [37]. In addition, some activated CD4^+^ T cells can directly kill β cells [38], while others enhance the cytotoxic activity of CD8^+^ T cells [39]. CD8^+^ T cells recognize GAD65-derived class I epitopes as well [5]. Notably, β cells expressing MHC class I molecules such as HLA-A*02:01 can present class I-restricted GAD65 peptides (*e.g.*, GAD65_114_–_123_), which are recognized by cytotoxic CD8^+^ T cells and can culminate in β-cell destruction [5].

However, to the best of our knowledge, we were the first to present a case of GAD65-associated autoimmune encephalitis and diabetes that did not exhibit the typical β-cell dysfunction seen in classic T1DM. Clarifying the type of diabetes in this patient was difficult. In this case report, the patient was initially diagnosed with diabetes in July 2023 at the age of 29. Approximately one month after the initial diagnosis, both fasting and 2-hour postprandial C-peptide levels were above 1 ng/mL. Eleven months later, the 2-hour postprandial C-peptide level remained above 1 ng/mL (Table **3**). This differs from classic T1DM, which typically exhibits low C-peptide levels in both fasting and stimulated states [40, 41]. Therefore, a C-peptide release test was performed using a 100-g steamed bun meal to further evaluate β-cell function. The test revealed a peak C-peptide level of 3.99 ng/mL, making the diagnosis of classic T1DM unlikely in this patient with GAD65-associated encephalitis.

Latent autoimmune diabetes (LADA), a unique form of diabetes that combines features of both type 1 and type 2 diabetes [42, 43], has the same spectrum of diabetes-associated autoantibodies as classic T1DM [42, 43], including GAD65 antibodies, pancreatic islet-cell antibodies (ICA), autoantibodies against insulin (IAA), insulinoma associated-2 autoantibodies (IA-2A), and zinc transporter-8 autoantibody (ZnT8A) [42]. Among them, GAD65 antibodies are recognized as one of the most sensitive immunological criteria for diagnosing LADA [44]. At the time of diagnosis, LADA patients release more insulin, exhibit slower β-cell deterioration during follow-up, and have a delayed need for insulin therapy compared to classic T1DM patients [42, 43]. Given the stimulated C-peptide levels of the steamed bun meal test (Table **3**), indicating relatively preserved β-cell function, and the positive GAD65 antibodies, this patient might be diagnosed with LADA. Of note, previous studies have indicated that the clinical phenotype of LADA varies depending on GADA titer levels [43]. LADA patients with high GADA titers display resemblances to those with classic T1DM, exhibiting a greater degree of insulin deficiency [43]. However, in the present case, despite persistently high GAD65 antibody titers (Fig. **1** and Table **2**), the patient did not develop the profound insulin deficiency typically seen in classic T1DM (Table **3**). This apparent paradox can be better understood in light of previous studies on the immunological role of GAD65 antibodies. Some studies suggest that GAD65 antibodies reflect the extent of T-cell-mediated immune responses [11]. The titer of GAD65 antibodies does not correlate directly with clinical phenotype or disease severity [11], which raises the possibility that GAD65 antibodies may merely serve as a bystander marker in some cases [6, 44]. Importantly, the clinical phenotype appears to be more closely determined by the specific antigenic epitopes targeted by GAD65 antibodies. For example, the presence of high-titer GAD65 antibodies in autoimmune limbic encephalitis does not necessarily imply concomitant T1DM [5]. Notably, previous studies have demonstrated that the epitopes recognized by GAD autoantibodies in patients with Stiff-Person Syndrome (SPS) and cerebellar ataxia differ markedly from those recognized in patients with limbic encephalitis or T1DM [32]. This distinction may help explain why, in the present case, despite high titers of GAD65 antibodies, the patient did not develop the profound insulin deficiency typically observed in classic T1DM (Table **3**).

However, this patient might have Type 2 Diabetes Mellitus (T2DM), as the GAD65 antibody is not only a biomarker for classic T1DM and LADA, but is also found in typical T2DM patients [44, 45]. Previous studies have suggested that some patients with GAD65 antibodies may develop T2DM [46]. For example, in the study by Niskanen *et al*., one-third of the non-diabetic participants who were GAD65 antibody positive at baseline developed T2DM 10 years later [47]. Similarly, GAD65 antibody positivity was strongly associated with incident T2DM in a non-diabetic adult population [48]. In our case, the patient had a longstanding history of seizures before being diagnosed with diabetes, similar to a previous report [21]. This prolonged diabetes-free period may be attributed to the extended subclinical immune response in the pancreas due to the presence of GAD65 antibodies, ultimately leading to the development of T2DM [49]. Moreover, high titers of antibodies to GAD65 were not predictive of the future development of insulin deficiency in adult T2DM [5, 50], suggesting that this patient may not necessarily require insulin therapy in the future.

## CONCLUSION

In summary, it is difficult to classify the types of diabetes in this case, as GAD65 positivity alone is insufficient to differentiate between LADA and T2DM. Therefore, it is essential to concurrently evaluate β-cell function and monitor its changes over time. Our case suggests to clinicians that, when GAD65-associated autoimmune encephalitis is accompanied by diabetes, greater attention should be paid to assessing and monitoring β-cell function. However, this report describes only a single patient, and thus the findings cannot be generalized. Further studies are warranted to explore this association in greater depth.

## Figures and Tables

**Fig. (1):**
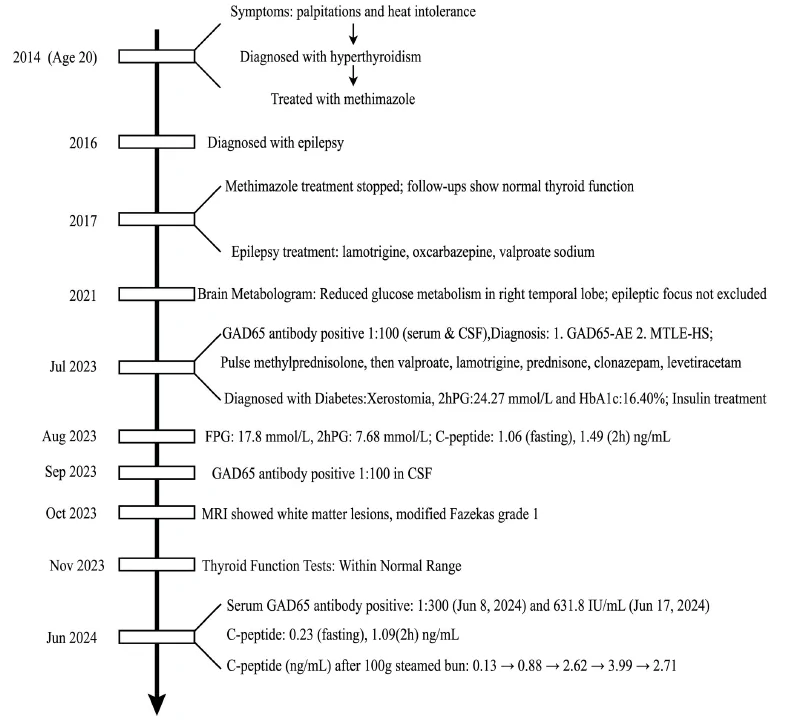
A timeline chart illustrating the chronological sequence of clinical and paraclinical findings. **Abbreviations:** GAD65: Glutamate decarboxylase 65; CSF: Cerebrospinal Fluid ; GAD65-AE: GAD65-associated autoimmune encephalitis; MTLE-HS: Mesial temporal lobe epilepsy with hippocampal sclerosis; 2hPG: 2-hour postprandial plasma glucose.

**Fig. (2):**
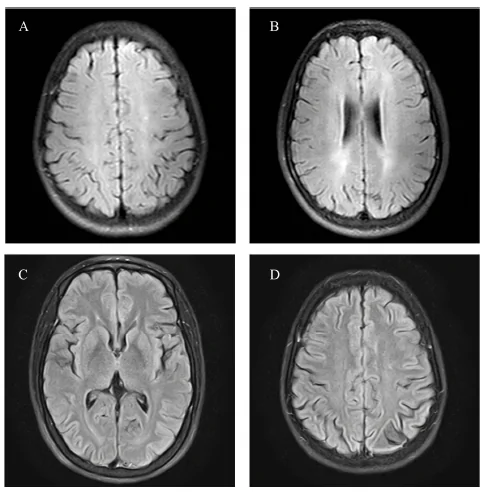
Cranial MRI scans of the patient. (**A** and **B**): The cranial MRI scan conducted on March 26, 2017, indicated a demyelinating lesion in the white matter of the left oval center. (**C** and **D**): The cranial MRI for the patient performed on October 26, 2023, revealed white matter lesions, classified as modified Fazekas grade 1.

**Fig. (3):**
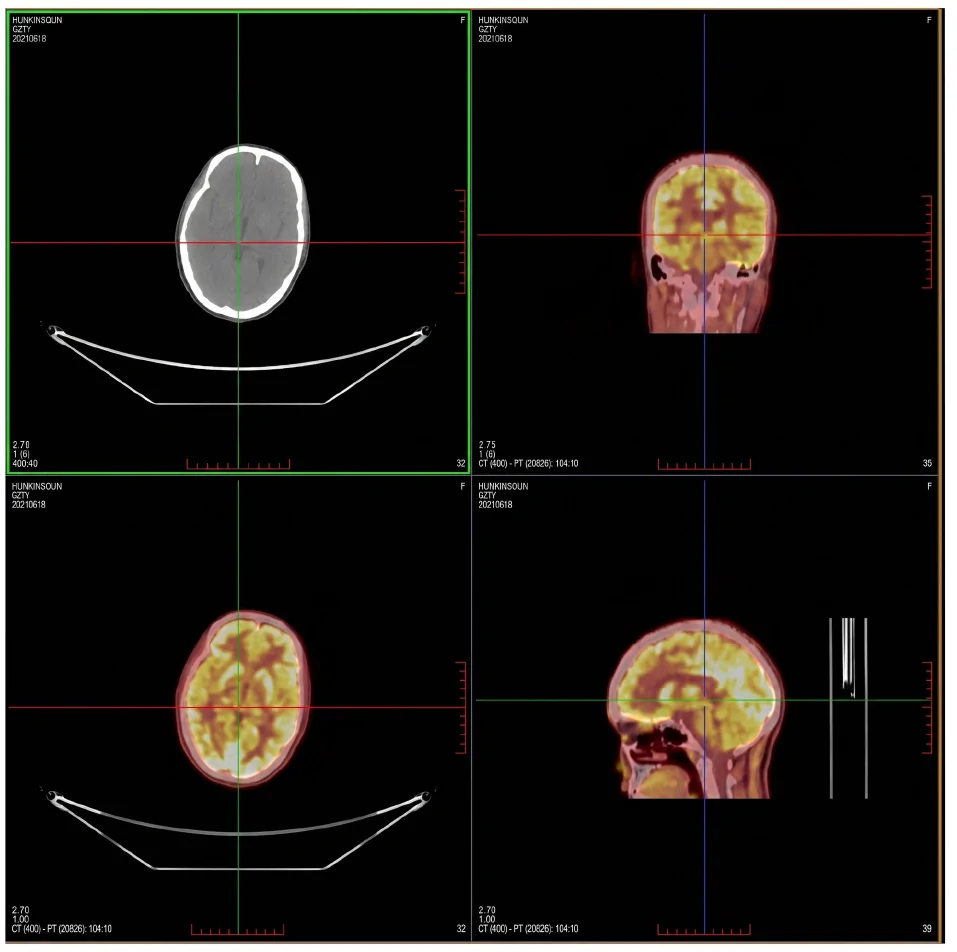
Brain Metabologram performed in 2021 showed reduced glucose metabolism in the right temporal lobe.

**Table 1:** Thyroid function test reports on November 28, 2023.

-	Result	Unit	Reference Range
T3	1.61	nmol/L	1.23-2.94
T4	81.74	nmol/L	65.64-154.44
FT3	4.02	pmol/L	3.08-6.50
FT4	12.49	pmol/L	9.65-21.88
TSH	1.53	mIU/L	0.4-5.86

**Table 2:** GAD65 antibody titers or concentrations of the patient.

Date	Serum	CSF
2023.07.17	1:100(CBA)	1:100(CBA)
2023.09.16	Not tested	1:100(CBA)
2024.06.07	1:300(CBA)	Not tested
2024.06.20	631.8IU/ml (Chemiluminescence, reference <10)	Not tested

**Abbreviations:** CSF: Cerebrospinal Fluid.

**Table 3:** The patient's blood glucose and C-peptide levels.

Date	-	0 h	0.5 h	1 h	2 h	3 h
2023.08.11*	Glucose (mmol/L)	17.80	-	-	7.68	-
	C-peptide (ng/mL)	1.06	-	-	1.49	-
2024.06.18*	Glucose (mmol/L)	6.48	-	-	4.52	-
	C-peptide (ng/mL)	0.23	-	-	1.09	-
2024.06.19^#^	Glucose (mmol/L)	6.02	6.35	14.91	18.16	15.64
	C-peptide (ng/mL)	0.13	0.88	2.62	3.99	2.71

**Note:** *Fasting and postprandial blood glucose and C-peptide measurement.

^#^Blood glucose and C-peptide levels after administering a standard 100-g steamed bun meal.

## Data Availability

The data and supportive information are available within the article.

## LIST OF ABBREVIATIONS

T1DM: Type 1 Diabetes Mellitus
Gad: Glutamate Decarboxylase
Mtle-Hs: Mesial Temporal Lobe Epilepsy With Hippocampal Sclerosis
CSF: Cerebrospinal Fluid
2hpg: 2-Hour Postprandial Plasma Glucose
LADA: Latent Autoimmune Diabetes

## STANDARDS OF REPORTING

CARE guidelines were followed.
